# Cantharidin downregulates PSD1 expression and inhibits autophagic flux in yeast cells

**DOI:** 10.1002/2211-5463.13196

**Published:** 2022-03-29

**Authors:** Swati Swagatika, Raghuvir Singh Tomar

**Affiliations:** ^1^ Laboratory of Chromatin Biology Department of Biological Sciences Indian Institute of Science Education and Research (IISER) Bhopal India

**Keywords:** autophagy, chloroquine, ethanolamine, GFP‐Atg8, Psd1

## Abstract

Cantharidin is a terpenoid compound of insect origin, naturally produced by male blister beetles as an antipredatory mechanism. Cantharidin has anticancer properties, which are attributed to its ability to induce cell cycle arrest, DNA damage, MAPK signaling pathway, and apoptosis. Cantharidin has been reported to induce apoptosis in triple‐negative breast cancer cells by suppressing autophagy via downregulation of Beclin 1 expression and autophagosome formation. However, it remains unclear which stage of the autophagic pathway is targeted by cantharidin. Herein, we report that yeast cells are sensitive to cantharidin, and external supplementation of ethanolamine (ETA) ameliorates the cytotoxicity. In addition, cantharidin downregulates phosphatidylserine decarboxylase 1 (PSD1) expression. We also report that cantharidin inhibits autophagic flux, and external administration of ETA could rescue this inhibition. Additionally, cotreatment with chloroquine sensitized the autophagy inhibitory effects of cantharidin. We conclude that yeast cells are sensitive to cantharidin due to inhibition of autophagic flux.

## Graphical abstract

Cantharidin has anticancer properties. Herein, we report that yeast cells are sensitive to cantharidin, and external supplementation of ethanolamine ameliorates the cytotoxicity. Cantharidin inhibits autophagic flux, and external administration of ethanolamine could rescue this inhibition. Cotreatment with chloroquine sensitized the autophagy inhibitory effects of cantharidin. We conclude that yeast cells are sensitive to cantharidin due to inhibition of autophagic flux.

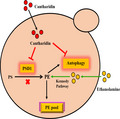

**Abbreviations**
CAN: cantharidin
CHO: choline
CQ: chloroquine
ETA: ethanolamine
GPI: glycosylphosphatidylinositol
HCQ: hydroxychloroquine
INO: inositol
PC: phosphatidylcholine
PE: phosphatidylethanolamine
PI: phosphatidylinositol
PS: phosphatidylserine
PSD1: phosphatidylserine decarboxylase 1
PSD2: phosphatidylserine decarboxylase 2

Cantharidin (CAN) is a terpenoid compound of insect origin with the Spanish fly, Cantharis vesicatoria, probably being the best‐known source. This molecule is naturally produced by the male blister beetles as an antipredatory mechanism and is presented to their female counterparts as a gift to defend their eggs from predators [1]. Since the time of its discovery in 1910 by a French chemist Robiquet, this molecule has been exploited to understand its mechanism of action. The anticancer properties of this compound are attributed to its ability to induce cell cycle arrest, DNA damage, MAPK signaling pathway, and apoptosis [2, 3, 4, 5]. Additionally, it is known to suppress cancer metastasis [6]. CAN has also been shown to be a potent inhibitor of protein phosphatase 2A (PP2A), a positive regulator of cell growth and division [7]. In yeast, it has been reported that cantharidin resistance gene 1 (CRG1) methylates CAN and renders it inactive [8]. It has also been reported to perturb the lipid homeostasis by altering short‐chain to long‐chain ratio of phosphatidylethanolamine (PE), phosphatidylserine (PS), and phosphatidylinositol (PI) species [8]. Recent findings suggest that CAN affects the GPI‐anchored protein sorting pathway by targeting CDC1‐mediated remodeling in yeast ER [9]. One report also reveals that CAN induces apoptosis in triple‐negative breast cancer cells by suppressing autophagy via downregulation of Beclin 1 (mammalian ortholog of yeast Vps30/Atg6) expression and autophagosome formation [10]. Although we have some knowledge about its role in autophagy inhibition, it remains unclear, however, which stage of autophagic pathway is being targeted by this molecule. Also, its other potential targets are yet to be explored.

Autophagy is a conserved cellular process by which organelles, cytoplasmic materials, and unwanted proteins are wrapped by a double‐membrane vesicle called autophagosome and are directed for degradation and recycling [11, 12, 13, 14]. This pathway has been very well studied in *Saccharomyces cerevisiae* and has been shown to be positively stimulated in response to nutritional stress like nitrogen‐ or carbon‐starved conditions or in the presence of rapamycin [15, 16, 17]. The initiation of autophagy is marked by the formation of a pre‐autophagosomal structure which later matures to form an isolation membrane. This is followed by a nucleation step wherein cargo like cytosolic proteins and organelles meant for degradation are selectively packaged onto this growing isolation membrane. This isolation membrane later expands and forms transient sequestering structures called as phagophores. Phagophore then matures into complete autophagosomes followed by its docking and fusion with the vacuoles allowing cargo delivery and degradation [18, 19, 20, 21, 22]. The formation and maturation of autophagosomes are a highly dynamic process and are orchestrated by several autophagy‐related (Atg) proteins [21, 23]. One such key protein involved in autophagosome biogenesis and its subsequent vacuolar fusion is Atg8 (LC3 in mammals) [24, 25]. Upon its translation, Atg8 is covalently conjugated to a lipid phosphatidylethanolamine (PE) at its C terminus exposed glycine residue in a series of ubiquitin‐like reactions to form Atg8‐PE [26]. This conserved Atg8 lipidation process requires several other Atg proteins and is extremely crucial as the PE acts as an anchor for autophagosome tethering and fusion to the vacuoles resulting in proper cargo delivery and autophagy [27, 28].

The PE for this lipidation process is derived from the total cellular PE pool [26]. PE along with PS, PC, and PI is the major phospholipids involved in the regulation of yeast lipid homeostasis [29, 30, 31]. PE is the second most abundant phospholipid found in mammalian cell membranes and is an important phospholipid in yeast [31]. PE in yeast is synthesized via four different pathways—by decarboxylation of PS to PE either (a) in the mitochondria by Psd1 enzyme or (b) in the Golgi/vacuoles by Psd2, (c) by exogenous supplementation of ethanolamine via Kennedy pathway, and (d) by acylation of lyso‐PE [32]. Out of these four pathways, PE biosynthesis by Psd1 contributes majorly (~ 70%) to the total PE pool inside the cells [33, 34]. This PE pool then contributes to several downstream consuming pathways like biogenesis of biological membranes, synthesis of PC from PE, glycosylphosphatidylinositol (GPI) anchoring, etc. [35]. PE has also been shown to positively regulate the autophagy process and longevity in yeast [29]. Additionally, yeast autophagy is reported to compete with other PE‐consuming downstream pathways for the total cellular PE pool [36].

With these premises, in the present study we have employed *Saccharomyces cerevisiae* as a model organism to investigate previously unknown genetic targets of CAN. Our initial viability assay revealed that wild‐type yeast cells are sensitive to CAN in a dose‐dependent manner, and external supplementation of only ETA, not other phospholipid precursors, was able to rescue this sensitive phenotype. Furthermore, our findings suggest that CAN targets PSD1 and probably decreases the total cellular PE pool, thus affecting its downstream PE‐consuming pathways. Also, autophagy is inhibited in the presence of CAN probably due to defects in autophagosome–vacuole fusion. Interestingly, exogenous supplementation with ETA was able to restore vacuolar delivery of cargo leading to functional autophagy. In addition, presence of chloroquine sensitized the autophagy inhibitory effects of CAN. Our investigation thus shows that CAN mediates *PSD1* downregulation and also inhibits autophagic flux.

## Materials and methods

### Strains, plasmids, chemicals, growth media, and growth conditions

The *Saccharomyces cerevisiae* strains used in this study are listed in Table S1. All yeast strains used in this study were grown in synthetic complete (SC) liquid media with 2% glucose at 30 °C, unless specified otherwise. SC liquid media was prepared by mixing all the basic components like yeast nitrogen base (YNB), ammonium sulfate (AS), and amino acids by following a standard protocol (Yeast Protocols Handbook, Clontech Laboratories, Inc.). The standard lithium acetate procedure was followed for yeast transformation and the resultant transformants were propagated in respective selective media [37]. For the preparation of solid agar media, 2% Bacto‐agar was added to SC liquid media. Plasmids used in this study are listed in Table S2. Media components, and all other reagents used in this study, were of molecular biology grade and purchased from Sigma‐Aldrich, Merck, GE Healthcare, HiMedia, Invitrogen, Bio‐Rad, New England BioLabs, and Thermo Fisher Scientific.

### Growth sensitivity and growth curve assays

Growth sensitivity assay (drop test or spot assay) was done by initially taking equal number of yeast cells (optical density; OD_600_ = 1.0) and then performing tenfold serial dilution (10^−1^, 10^−2^, 10^−3^, 10^−4^). 3 μL of each dilution was spotted onto solid SC‐agar plates with or without treatment (s). All the plates were incubated at 30 °C (or 37 °C) and growth of yeast strains was recorded after 72 h by scanning the plates using a HP scanner.

For liquid media growth curve analysis, equal number (OD_600_ = 0.1) of exponentially growing yeast cells was treated with either DMSO (control) or indicated doses of the compounds in duplicate manner and then seeded onto a 96‐well cell culture plate. Growth curves were analyzed for each treatment using OD_600_ values measured at a regular interval of 30 min for a time period of 48 h using a plate reader (Eon™ Microplate Spectrophotometer).

### RNA isolation, cDNA synthesis, and RT‐PCR

Total cellular RNAs were extracted from yeast cells by using a heat/freeze phenol method as described earlier [38]. 1 μg of pure RNA was reverse‐transcribed to cDNA by using iScript cDNA Synthesis Kit (Bio‐Rad, Catalog 1708891). The cDNA was then used to put RT‐PCR as previously described [39]. Primers listed in Table S2 were used to check the mRNA expression levels of PSD1 and CRG1 genes relative to the levels of ACT1. The data obtained were then analyzed using the $2^{‐\Delta \Delta C_{T}}$ method and plotted onto graph using graph pad prism.

### Isolation of yeast protein extracts

Yeast whole‐cell protein extracts were isolated by following 20% trichloroacetic acid (TCA) precipitation method as described previously [40]. Briefly, equal number of cells was harvested and washed once with 20% of TCA. Cell pellets were then resuspended in 20% of TCA and cell lysis was done by adding glass beads and vortexing the samples rigorously for 10 min. Post‐centrifugation, the pellet was washed once with 0.5 m of Tris/Cl (pH 7.5) and then resuspended in 0.5 m Tris/Cl and SDS loading buffer. The sample was boiled at 100 °C for 5 min and centrifuged at maximum speed for 15 min to remove the debris. The supernatant was collected for western blot analysis.

### Western blot analysis

The protein extracts were resolved by running SDS/PAGE and then transferring the protein to nitrocellulose/PVDF membranes following standard electrophoresis methods. Post‐transfer, the membranes were blocked for 1 h using blocking buffer (2.5–3% bovine serum albumin in TBST; Tris buffer saline containing 0.05% Tween 20) followed by incubation with primary antibody for 90 min. After washing with TBST thrice, the membranes were incubated with respective secondary antibody for 45 min. The blots were washed again with TBST thrice, left for air‐drying, and scanned by using Odyssey infrared imager (LI‐COR Biosciences). Following primary antibodies were used in this study: anti‐GFP (Sigma, Catalog G1544), anti‐Atg8 (gift from Dr. Yoshinori Ohsumi), and anti‐Psd1 (gift from Dr. Thomas Becker). Polyclonal antibodies against recombinant Tbp and Rap1 were raised in rabbit. Goat anti‐rabbit (Invitrogen, Catalog A32734) secondary antibody was used. To check Atg8 lipidation, protein samples were run in 6 m urea‐containing SDS/PAGE (18%) and PVDF membrane was used for transfer.

### Fluorescence imaging

For this, exponentially growing wild‐type yeast cells (Vph1 mcherry or GYS638 or *ypt7Δ/Δ* strains) carrying the GFP‐ATG8 plasmid were treated with DMSO (control) or indicated doses of CAN for 6, 12, or 18 h. Post‐treatment cells were harvested at respective time points, washed twice with 1X phosphate buffer saline (PBS; pH 7.4), and imaged using 100× oil‐immersion objective lens of ZEISS ApoTome.2 microscope. The subcellular localization of Gfp‐Atg8/ mRFP‐APE1 fusion protein was then monitored. Further all the images were processed using ZEN‐2012 software.

### Statistical analysis

All data are representative of 3 independent experiments and are presented as mean ± standard error mean (SEM). The significance of differences between sets of data was determined by Student’s *t*‐test; ns denotes *P* > 0.05, * denotes *P* ≤ 0.05, and ** denotes *P* ≤ 0.01 (as per the graph pad prism).

## Results

### CAN inhibits yeast cell growth in a dose‐dependent manner

**Fig. 1:**
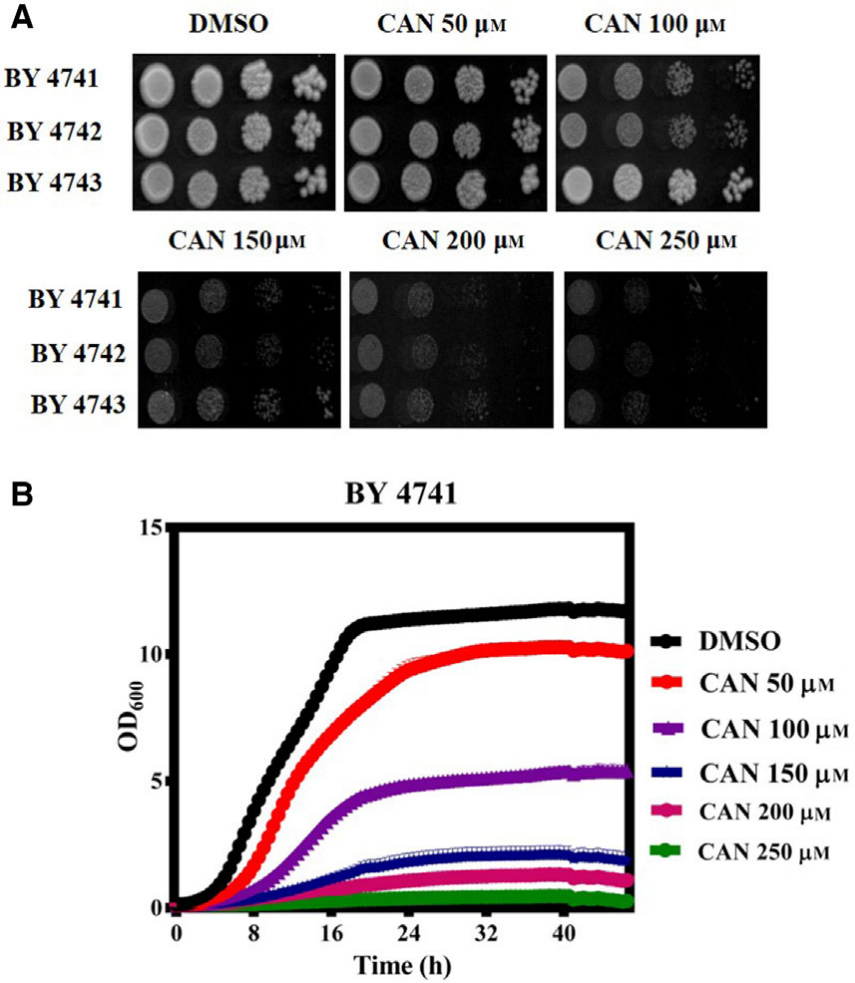
Wild‐type yeast cells show sensitivity to CAN. (A) Yeast serial drop test assay. Yeast saturated cultures of wild‐type cells were 10‐fold serially diluted (10^−1^, 10^−2^, 10^−3^, 10^−4^) in 1.0 mL of sterile double‐distilled water and 3 µL of each dilution was spotted on SCA plates containing DMSO (control) or CAN (50, 100, 150, 200, and 250 µm). All plates were incubated at 30 °C for 72 h and scanned. (B) Growth curve assay: exponentially growing yeast cultures of the wild‐type strain BY4741 were treated with either DMSO (control) or indicated doses of CAN and the growth was monitored by measuring OD_600_ at regular intervals of 30 min for 48 h using a plate reader.

We initially determined the effective dose of CAN in solid agar media by performing spot assay and liquid media by growth curve analysis. For the spot assay, overnight grown wild‐type yeast strains (BY4741, BY4742, BY4743) were spotted onto agar plates containing increasing concentrations of CAN (50, 100, 150, 200, and 250 μm) and incubated at 30 °C for 48 h (Fig. 1A). For liquid medial growth curve analysis, exponentially grown wild‐type yeast cells were treated with indicated CAN doses and allowed to grow for ~ 48 h in a 96‐well plate reader (Fig. 1B). In both spot assay and liquid media growth curve analysis, we observed a similar pattern of dose‐dependent decrease in yeast cell viability.

### Ethanolamine supplementation remediates the cytotoxic effects of CAN

**Fig. 2:**
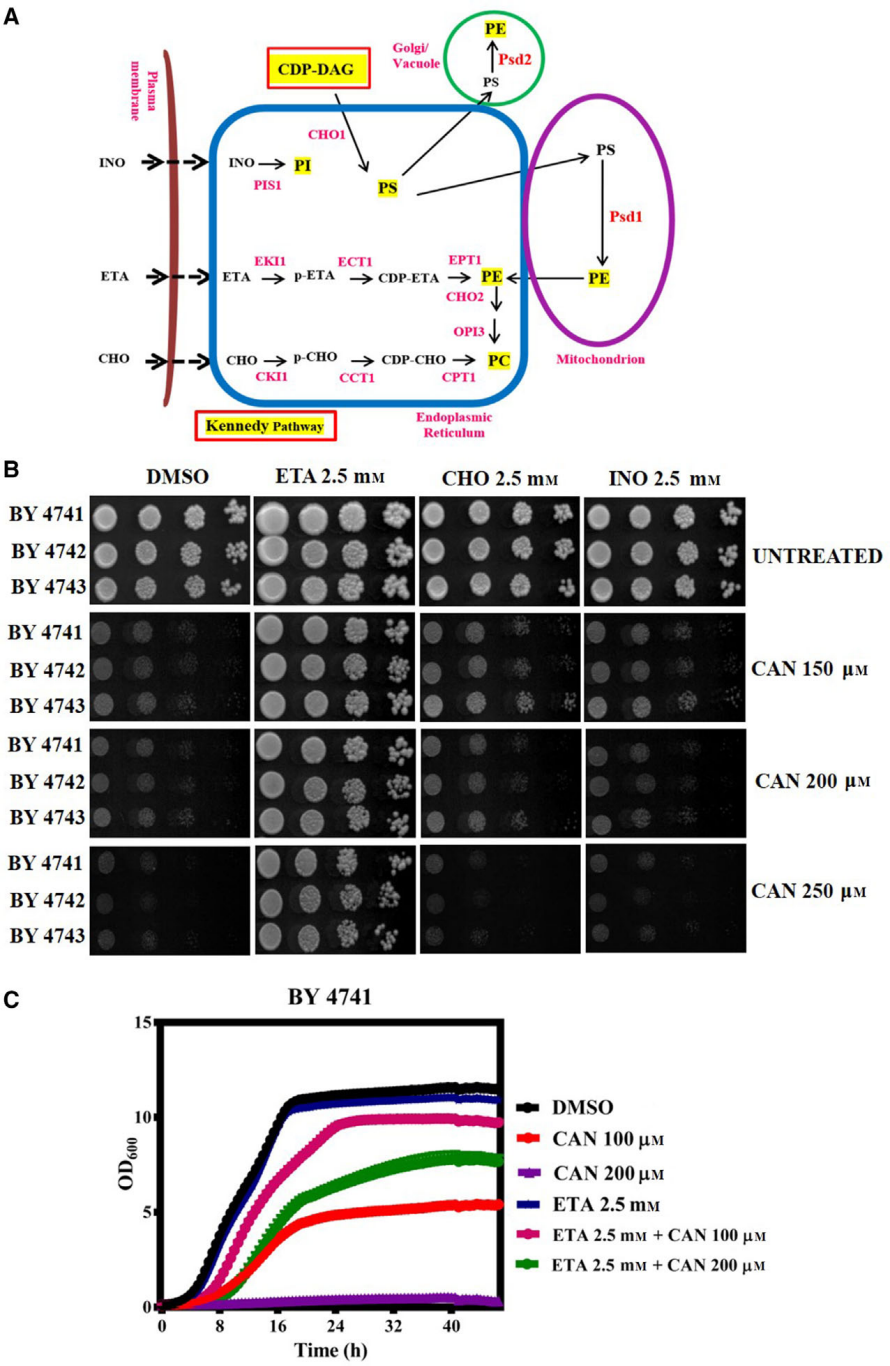
ETA supplementation rescued the CAN sensitivity. (A) Schematic representation of CDP‐DAG and Kennedy pathway in yeast phospholipid metabolism. External supplementation of ETA bypasses the conventional CDP‐DAG pathway and utilizes the Kennedy pathway for the synthesis of phosphatidylethanolamine inside the cells. (B) Yeast spot assay. Yeast saturated cultures of wild‐type cells were 10‐fold serially diluted (10^−1^, 10^−2^, 10^−3^, 10^−4^) in 1.0 mL of sterile double‐distilled water, and 3 µL of each dilution was spotted on SCA plates containing DMSO (control) or CAN (150, 200, and 250 µm) or ETA/CHO/INO (2.5 mm) in alone or in combinations. All plates were incubated at 30 °C for 72 h and photographed. The representative ETA 2.5 mm spotting assay image is identical in Figs 2B and 3B as we had performed both these spot assay experiments at the same time and this plate was kept common. (C) Growth curve assay: exponentially growing yeast culture of the wild‐type strain BY4741 was treated with either DMSO (control) or CAN (100 and 150 µm) or ETA 2.5 mm in alone or in indicated combinations and the growth was monitored by measuring OD_600_ at regular intervals of 30 min for 48 h using a plate reader.

As wild‐type yeast cells were sensitive to CAN in a dose‐dependent manner, our next obvious question was to reason for this CAN‐induced sensitivity. In literature, CAN is known to perturb the cellular lipid homeostasis [8]. Lipid homeostasis in yeast is governed majorly by four phospholipids namely phosphatidylethanolamine (PE), phosphatidylcholine (PC), phosphatidylinositol (PI), and phosphatidylserine (PS) [41]. These phospholipids are synthesized mainly via two de novo pathways—CDP‐DAG pathway and the Kennedy pathway [42, 43] (Fig. 2A). In addition, external supplementation of phospholipid precursors has been shown to restore the respective phospholipids inside the cells [44, 45]. In this line, we wanted to check whether CAN‐induced perturbation in lipid homeostasis is responsible for the sensitive phenotype and whether it is so whether external supplementation of the precursors of these major phospholipids can rescue this sensitivity. For this, we performed spot assay with wild‐type strains in plates containing both CAN and the precursors of major phospholipids like ethanolamine (ETA), choline (CHO), and inositol (INO). Spot assay data revealed external administration of ETA could rescue CAN‐induced sensitive phenotype completely, whereas CHO and INO supplementation showed little to no rescue (Fig. 2B). We also cannot ignore the fact that external administration of ETA in general influences the yeast cell growth and proliferation [29]. We did use less ETA, that is, ETA 1 mm initially for the spot assay experiment (Fig. S1). We observed that compared with ETA 1 mm, ETA 2.5 mm showed significantly better rescue against CAN‐mediated toxicity. Also, in case of only ETA‐treated plates there was hardly any difference between the growth of cells in presence of ETA 1 and 2.5 mm. So, in line with previous reports where a range of ETA concentrations from 2 to 50 mm has been used, and based on our growth phenotype assays, we decided to use ETA 2.5 mm for our rescue experiments [29, 46]. We further validated the ETA‐mediated rescue in liquid media growth curve analysis as well (Fig. 2C). We thus concluded that CAN‐mediated cytotoxicity is ameliorated in the presence of ETA.

### CAN perturbs yeast membrane integrity

**Fig. 3:**
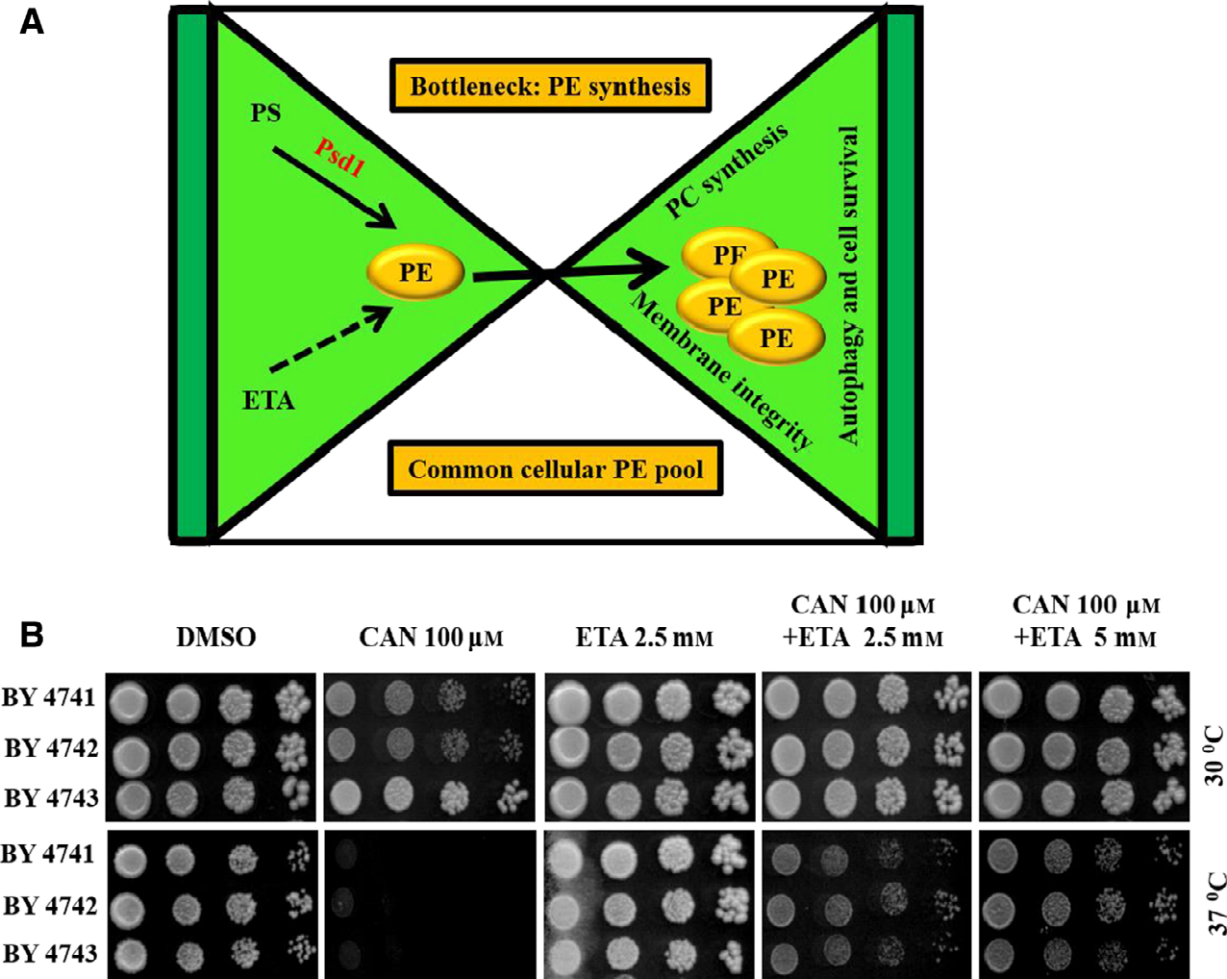
ETA supplementation rescued the CAN‐induced temperature sensitivity at 37 °C. (A) PE biosynthesis is the bottleneck for CAN tolerance. The PE that is synthesized inside the cell with the help of Psd1 or PE derived from externally supplemented ETA contributes to the total cellular PE pool. This PE is then channelized toward several other downstream PE‐consuming pathways. (B) Yeast spot assay was performed with wild‐type yeast cells on SCA plates supplemented with DMSO (control) or CAN (100 µm) or ETA (2.5 or 5 mm) in alone or in indicated combinations. All plates were incubated at 30 and 37 °C for 72 h and photographed. The representative ETA 2.5 mm spotting assay image is identical in Figs 2B and 3B as we had performed both these spot assay experiments at the same time and this plate was kept common.

As only ETA supplementation was able to rescue the CAN‐mediated cytotoxicity, we next hypothesized that cellular PE availability might be crucial for CAN tolerance. The total PE pool inside the cell serves as the bottleneck for all the major downstream cellular PE‐consuming pathways—membrane integrity, GPI anchoring of cell wall proteins, PC synthesis, Atg8 lipidation for functional autophagy, etc. [36, 47] (Fig. 3A). So, in an attempt to investigate whether CAN perturbs the cellular PE homeostasis or not, we checked the effect of CAN in these downstream pathways. Our hypothesis was, if CAN is indeed mediating its cytotoxic effects by targeting the cellular PE pool, we might see its perturbing effects in the downstream PE‐consuming pathways as well. One of such downstream PE‐consuming pathways that we first took into consideration was yeast membrane lipid integrity. PE is a major phospholipid component of cell membranes in eukaryotic organisms and along with PC accounts for > 50% of the total phospholipid species in eukaryotic membranes [30, 48, 49]. PE plays a major role in the structure and function of these membranes and is essential to the viability of the cell [50]. Growing yeast cells at higher temperature of 37 degrees is known to disrupt its membrane lipid integrity. With this entire premise, we performed spot assay of wild‐type yeast cells in presence of a sublethal dose of CAN and allowed the cells to grow at both 30 degrees (the recommended yeast cell growth condition) and 37 degrees. Post‐72 h of incubation, we found that the presence of higher temperature potentiated the toxic effects of CAN and ETA supplementation could rescue this hypersensitivity (Fig. 3B). This finding hints at the role of CAN in perturbation of yeast membrane lipid integrity.

### CAN downregulates the PSD1 expression levels

**Fig. 4:**
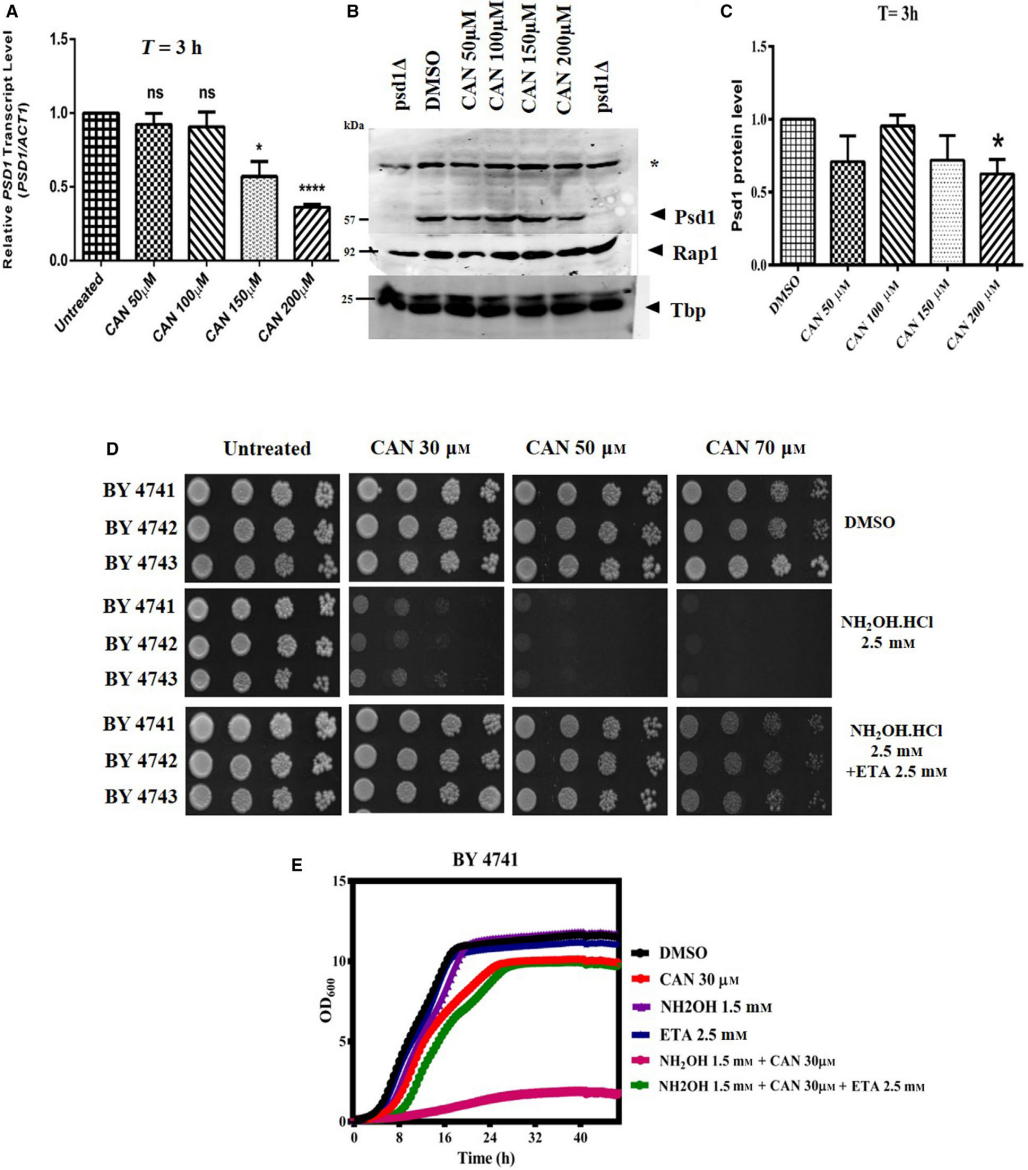
CAN inhibits *PSD1* expression. (A) Real‐time RT‐PCR quantification of PSD1 gene expression. Exponentially growing wild‐type yeast cells (BY4741) were treated with DMSO (control) or increasing doses of CAN (50, 100, 150, and 200 µm) and allowed to grow for 3 h. Post‐treatment cells were harvested, RNA–isolated, and cDNA prepared. RT‐PCR analysis of PSD1 gene relative to the levels of ACT1 mRNA was performed using the $2^{‐\Delta \Delta C_{T}}$ method and plotted onto graph. (B) Cells harvested from above experiment were also subjected to protein isolation followed by western blot analysis using anti‐Psd1 antibody. *psd1Δ* is used as a negative control. Tbp and Rap1 are used as loading controls. (C) Quantification of Psd1 protein levels post‐CAN treatment using Tbp as a loading control (D) Supplementation with NH_2_OH.HCl potentiated the cytotoxic effects of CAN and presence of ETA could rescue this phenotype. Yeast serial drop test assay was performed. Yeast saturated cultures of wild‐type cells were 10‐fold serially diluted (10^−1^, 10^−2^, 10^−3^, 10^−4^) in 1.0 mL of sterile double‐distilled water and 3 µL of each dilution was spotted on SCA plates containing DMSO (control) or CAN (30, 50, and 70 µm) or NH_2_OH.HCl (2.5 mm) or ETA (2.5 mm) in alone or in indicated combinations. All plates were incubated at 30 °C for 72 h and photographed. (E) Growth curve assay: exponentially growing yeast culture of the wild‐type strain BY4741 was treated with either DMSO (control) or CAN (30 and 50 µm) or NH_2_OH.HCl (1.5 mm) or ETA (2.5 mm) in alone or in indicated combinations and the growth was monitored by measuring OD_600_ at regular intervals of 30 min for 48 h using a plate reader. All data are representative of 3 biologically independent experiments and are presented as mean ± standard error mean (SEM). The significance of differences between sets of data was determined by Student’s *t*‐test; ns denotes *P* > 0.05, * denotes *P* ≤ 0.05, and ** denotes *P* ≤ 0.01 (as per the graph pad prism).

PE pool inside the cells is mostly maintained via the Psd1‐mediated decarboxylation of PS or via exogenous supplementation of ETA [32]. Psd1p of mitochondria contributes majorly to the cellular PE pool [51, 52]. In line with these observations and previous finding which shows that PSD1 shows synthetic lethality with CRG1 and increased sensitivity toward CAN [9], we reasoned that CAN might be targeting PSD1 for exhibiting its toxicity. In this regard, we allowed the wild‐type yeast cells to grow in the presence or absence of increasing doses of CAN (50, 100, 150, and 200 µm) for 3 h. RT‐PCR analysis revealed that the gene expression level of PSD1 decreased significantly post‐CAN treatment at higher doses (CAN 150 and 200 µm; Fig. 4A). We also performed RT‐PCR analysis for CRG1, a gene already reported to be upregulated post‐CAN treatment [8], just to confirm that the pattern of gene expression that we got in case of *PSD1* is unique for that gene and is not just an artifact (Fig. S2). The cells harvested from above experimental setup were also subjected to protein extraction followed by western blot analysis using anti‐Psd1 antibody [51]. We could not observe major reduction of Psd1 protein at lower doses but significant reduction could be seen at higher dose of CAN 200 µm (Fig. 4B,C). To further strengthen our data, we performed spot assay with external supplementation of hydroxylamine hydrochloride. A reagent previously shown to affect the enzymatic activity of phosphatidylserine decarboxylase *in vitro* [53]. Our hypothesis was if CAN is mediating its cytotoxicity by inhibiting PSD1 expression then supplementation of hydroxylamine should further contribute to this cytotoxicity resulting in hypersensitive phenotype. As expected, yeast cells showed hypersensitivity in presence of hydroxylamine (1 mm) at a very sublethal dose of CAN 30 µm and this hypersensitivity could be rescued by supplementing the media with ETA 2.5 mm (Fig. 4D). We also obtained similar results in liquid media growth curve analysis (Fig. 4E). These findings suggest that hydroxylamine potentiated the CAN‐mediated sensitivity which could be overcome in presence of ETA. We further substantiated this finding by performing liquid media growth curve analysis using another PSD1 inhibitor, DOX. Our data revealed that presence of DOX rendered the cells more sensitive toward CAN and the observed degree of sensitivity is less when compared to hydroxylamine hydrochloride (Fig. S3). We concluded that ETA when externally supplemented is able to bypass the requirement of Psd1 and alternatively contributes to PE synthesis via Kennedy pathway which in turn mitigates CAN stress (Fig. 2C).

### PSD1 is a potential genetic target of CAN

**Fig. 5:**
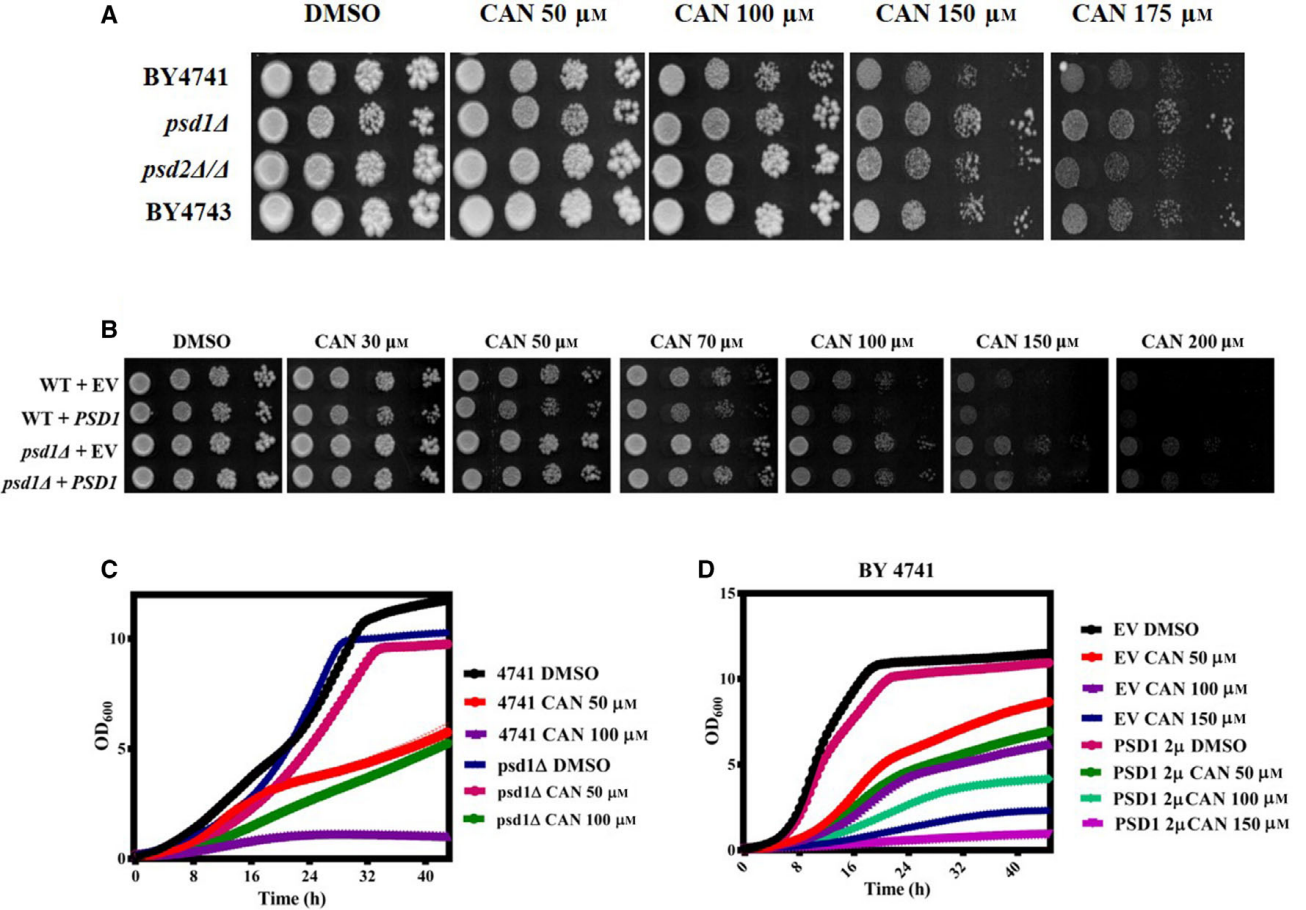
Effect of CAN on *psd1Δ* cells. (A) Yeast spot assay. Yeast saturated cultures of mutants *psd1Δ* and *psd2Δ/Δ* along with their respective wild types BY4741 and BY4743 were 10‐fold serially diluted (10^−1^, 10^−2^, 10^−3^, 10^−4^) in 1.0 mL of sterile double‐distilled water and 3 µL of each dilution was spotted on SCA plates containing DMSO (control) or CAN (50, 100, 150, and 200 µm). All plates were incubated at 30 °C for 72 h and photographed. (B) Yeast spot assay was performed using BY4741 and *psd1Δ* strains transformed with an empty vector or a 2 µ multicopy PSD1 plasmid in presence of DMSO or indicated doses of CAN. (C, D) Growth curve assay: exponentially growing wild‐type yeast strains (transformed or untransformed) and mutants were subjected to growth curve analysis in presence or absence of CAN and the growth was monitored by measuring OD_600_ at regular intervals of 30 min for ~ 42 h using a plate reader.

As our previous findings reveal that CAN reduced the Psd1 levels, we next wanted to investigate whether *PSD1* is one of its previously unidentified genetic targets. To test this, we performed a spot assay analysis wherein we spotted the overnight grown *psd1Δ* and *psd2Δ/Δ* strains along with their respective wild‐type strains onto agar plates containing increasing doses of CAN (50, 100, 150, and 175 µm). Post‐48‐h incubation, we observed that deletion of *PSD1* made the cells resistant toward the cytotoxic effects of CAN as compared to its wild type (Fig. 5A). On the other hand, the deletion of *PSD2* (a gene encoding for a PE synthesizing enzyme localized to Golgi/vacuole and contributing only a little toward the PE pool) rendered the cells equally sensitive as that of its respective wild type. We obtained the similar results in liquid media growth curve analysis also (Fig. 5C). As *psd1Δ* cells showed better growth than wild‐type cells, we next investigated whether lack of *PSD1* is being compensated by *PSD2* or *CRG1*. In this direction, qRT‐PCR analysis was performed and relative mRNA levels of these genes in both WT and *psd1Δ* cells before and after CAN treatment was compared. No significant difference in the gene expression level could be seen (Fig. S4).

Next, we attempted to rescue the CAN‐mediated cytotoxicity by transforming the cells with a 2 µ multicopy plasmid expressing 20–30 copies of *PSD1*. In this direction, we allowed the WT and *psd1Δ* cells either transformed with an empty vector or a PSD1 2 µ plasmid to grow in presence or absence of increasing doses of CAN. Surprisingly, instead of showing rescue phenotype, the presence of more copies of *PSD1* rendered the cells hypersensitive toward CAN as compared to that of untransformed ones (Fig. 5B,D). In line with previous reports that suggests not all drugs affecting the gene are resisted by target overexpression, we also believe that CAN in addition to reducing its target expression is additionally inducing some kind of target‐catalyzed toxic reaction that is proving lethal to the cells. Because of this, target (PSD1) overexpression enhanced CAN sensitivity instead of conferring resistance. One of the other possible scenarios could be that lack of PSD1 activity causes hormetic effect. So, under CAN stress, PSD1 when present in low levels is able to stimulate the growth of cells but when present in abundance is proving detrimental. This particular aspect is highly intriguing and needs additional experiments to be proved.

### CAN inhibits autophagy

**Fig. 6:**
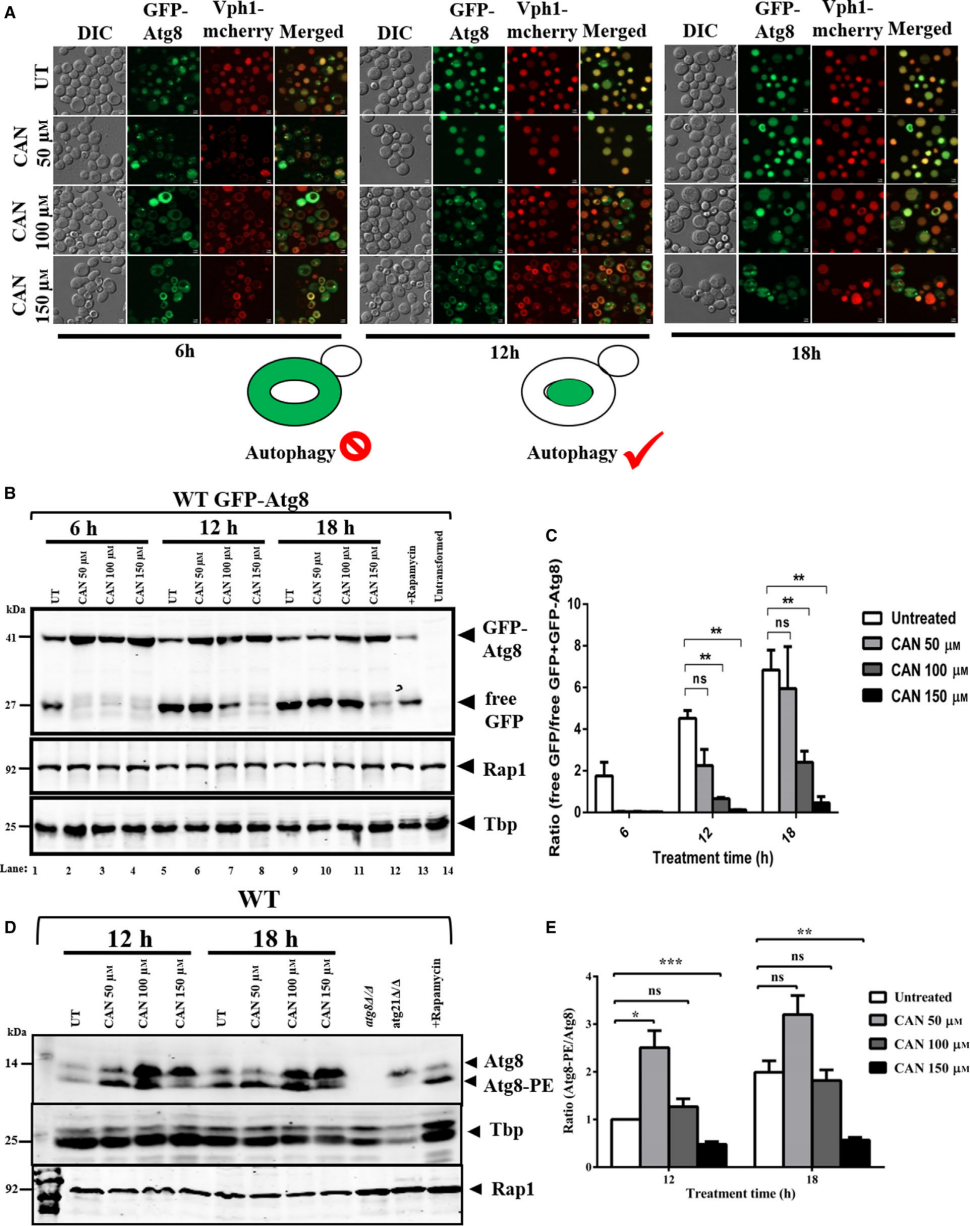
CAN‐treated cells were defective for autophagy. (A) CAN inhibits the delivery of GFP‐Atg8 fusion protein to the vacuoles. Exponentially growing wild‐type yeast cells (BY4741 Vph1 mcherry strain) carrying the GFP‐ATG8 plasmid were subjected to DMSO (control) or CAN‐treated conditions (50, 100, and 150 µm) for 6, 12, and 18 h. Cells were harvested at respective time points and processed for fluorescence microscopy. The subcellular localization of Gfp‐Atg8 fusion protein was then assessed using EGFP green filter‐ λex = 450–490 nm, λem = 500–550 nm. Vph1‐mCherry protein was used to mark the vacuolar membrane using mCherry filter. In a functional autophagic pathway, the GFP fluorescence is initially seen in the cytosol but later (during the completion stage) shifts to the vacuole after the transportation of GFP‐Atg8 into it. Note that under CAN 150 µm treated conditions, the GFP‐Atg8 fusion protein is present near the PAS even after 18 h post‐treatment. Representative images from three independent experiments are shown. Scale bar represents 2 μm. (B) Immunoblot for GFP‐Atg8 processing assay. Vacuolar delivery of GFP‐Atg8 fusion protein was assessed by western blotting using an anti‐GFP antibody. The strains, treatment, and growing conditions were same as described in (A) above. Samples were processed using TCA precipitation method and resolved using 12% SDS/PAGE. As GFP is resistant to vacuolar degradation, completion of autophagy is marked by the accumulation of free GFP in the vacuole. Note that with increase in CAN concentration, there is less/no accumulation of free GFP in the vacuoles. Rap1 and Tbp served as loading controls. Rapamycin (100 ng·mL^−1^ for 6 h) treated and untransformed cells were used as positive and negative controls, respectively. Representative images from three independent experiments are shown here. (C) Quantification of GFP‐Atg8 processing assay. The ratio between free GFP and GFP‐Atg8 fusion protein bands was quantified using imagej software and used as a readout for autophagic flux. Results from three independent experiments are expressed as the mean ± SEM. Results were compared using Student’s t‐test where ns denotes *P* > 0.05 and ** denotes *P* ≤ 0.01. (D) WT BY4741 cells were harvested after treatment with CAN for 12 and 18 h. The cells were then subjected to protein isolation followed by western blot analysis using anti‐Atg8 antibody to check for the levels of lipidated and unlipidated form of Atg8. (E) Quantification of Atg8 lipidation assay.

As CAN significantly inhibited the expression of PSD1 in wild‐type cells and PSD1 expression is directly correlated to the downstream PE‐consuming pathways, we next investigated the role of macroautophagy (one of the major PE‐consuming pathways inside the cell) in CAN tolerance [36]. Macroautophagy is responsible for the degradation and recycling of cytosolic components. It selectively removes the harmful substances and mobilizes the nutrients under starvation conditions [19]. This pathway is initiated by the formation and maturation of autophagosome, followed by Atg8‐mediated cargo recruitment, its conjugation with PE, and autophagosome formation. At the end, the autophagosome gets fused with the vacuole to deliver the cargo for degradation and recycling (Fig. S5). We used GFP‐ATG8 as a tool to monitor macroautophagy [54]. When autophagy is induced, GFP‐Atg8 is transported to the vacuoles from cytosol. We performed this experiment by allowing the wild‐type cells harboring GFP‐ATG8 (autophagy marker) and VPH1 mcherry (vacuolar membrane marker) plasmids to grow in the presence of CAN 50, 100, and 150 µm for 6, 12, and 18 h. Our fluorescence microscopic analysis revealed CAN‐mediated inhibition of cargo delivery by GFP‐Atg8 fusion protein to the vacuoles (Fig. 6A). At CAN 50 µm dose, the GFP fluorescence could be seen in the cytosol for initial 6 h which later (12 h) gets localized to the vacuole completely thus marking the completion of autophagy. At CAN 100 µm dose, the GFP fluorescence is localized to the cytosol for the initial 12 h and later gets shifted to the vacuole. At CAN 150 µm dose, the GFP fluorescence could be seen in the cytosol for all the time points taken. So, with increase in CAN dose there was concomitant decrease in the ability of the GFP‐Atg8 fusion protein to deliver the cargo to the vacuoles.

To further validate this finding, we performed GFP‐Atg8 processing assay to monitor the vacuolar delivery of GFP‐Atg8 fusion protein by western blot analysis using anti‐GFP antibody [54]. The completion of autophagy is marked by the successful delivery of GFP‐Atg8 fusion protein carrying the cargo to the vacuoles. Atg8 being an endogenous protein is susceptible to degradation by vacuolar hydrolases whereas GFP being an exogenous protein remains resistant. So, the amount of free GFP detected is a direct readout of the autophagic flux happening inside the cells. Our western blot analysis revealed the decrease in the amount of free GFP and hence autophagic flux upon CAN treatment (Fig. 6B). As expected, with increase in the CAN concentration, there is decrease in the ratio of free GFP to GFP‐Atg8 fusion protein (Fig. 6C). Also, the western blot analysis data followed a very similar trend to the one we had seen in microscopic analysis. Interestingly, we also observed increased protein levels of Atg8 in CAN‐treated cells compared with the untreated cells. Also, based on our western blot and microscopy analysis, we can conclude that lower doses of CAN delays whereas higher doses of CAN block the autophagic flux. Because of delay in autophagic flux, we can see the slow release of free GFP which corresponds to the appearance of free GFP band only after 12 h of yeast cells exposure to CAN 50 and 100 µm.

Next, we validated our findings by Atg8 lipidation assay, a useful method for assessing the autophagic activity. Atg8 lipidation anchors the protein to autophagosomal membrane and is crucial for autophagosome biogenesis, followed by its tethering to vacuole and cargo delivery [22, 26]. With this premise, we next examined the level of Atg8 lipidation in CAN‐treated cells by urea/SDS/PAGE western blot. The lipidated form of Atg8 (Atg8‐PE) being hydrophobic in nature tends to migrate faster than nonlipidated Atg8 and antibody directed against Atg8 can detect the shift in both the forms of protein [54]. We found that the level of lipidated Atg8 (Atg8‐PE) was markedly less in CAN 150 µm treated cells as compared to the control (Fig. 6D,E). Above findings thus indicate that CAN inhibits autophagic flux.

### External administration of ETA ameliorates CAN‐mediated autophagy inhibition

**Fig. 7:**
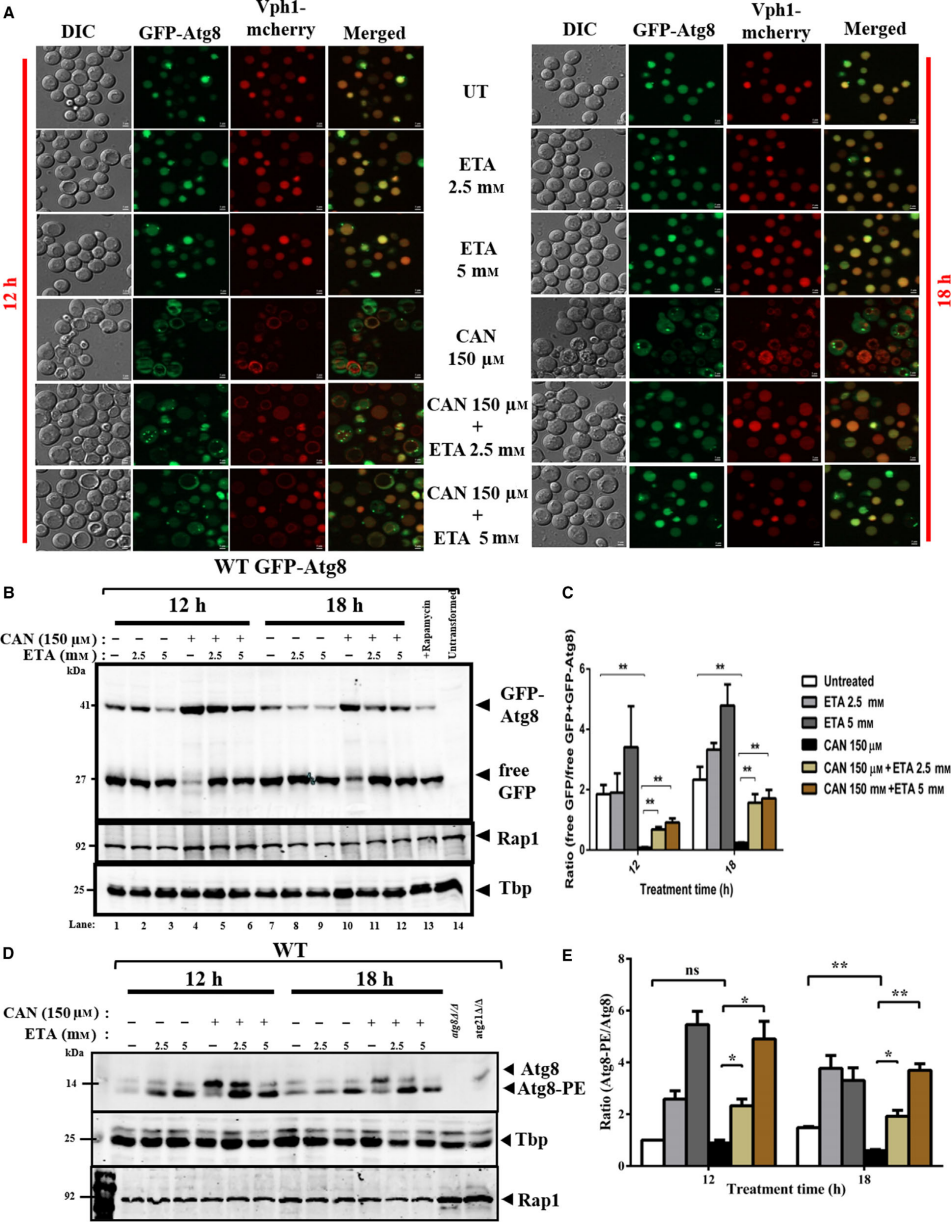
ETA supplementation remediated CAN‐mediated autophagy inhibition (A) Supplementation with ETA facilitated the delivery of Gfp‐Atg8 fusion protein to the vacuoles in yeast. Exponentially growing wild‐type yeast cells carrying the GFP‐ATG8 plasmid were treated with DMSO (control) or CAN (150 µm) or ETA (2.5 and 5 mm) in alone or in indicated combinations for 12 and 18 h. The subcellular localization of Gfp‐Atg8 fusion protein was then assessed via fluorescence microscopy using EGFP green filter. ETA supplementation neutralized the autophagy inhibition by CAN and favored the transportation of GFP‐Atg8 fusion protein from cytosol to vacuoles. This is marked by the GFP fluorescence seen inside the vacuoles. Representative images from three independent experiments are shown. Scale bar represents 2 μm. (B) Immunoblot for GFP‐Atg8 processing assay. Vacuolar delivery of GFP‐Atg8 fusion protein was assessed by western blotting using an anti‐GFP antibody. The strains, treatment, and growing conditions were same as described in (A) above. ETA supplementation favored the transportation of GFP‐Atg8 to the vacuoles which is marked by increased amount of free GFP. Representative images from three independent experiments are shown here. (C) Quantification of GFP‐Atg8 processing assay. The ratio between free GFP and GFP‐Atg8 fusion protein bands was quantified using imagej software and used as a readout for autophagic flux. Results from three independent experiments are expressed as the mean ± SEM. Results were compared using Student’s *t*‐test where ns denotes *P* > 0.05 and ** denotes *P* ≤ 0.01. (D) Presence of ETA facilitated Atg8 lipidation even in the presence of CAN. Western blot analysis was performed to check the levels of lipidated and unlipidated Atg8 post‐cotreating the cells with CAN and ETA for indicated time points. (E) Quantification of Atg8 lipidation assay.

As external supplementation of ETA could rescue CAN‐mediated cytotoxicity, we next went on to check whether it is able to remediate autophagy inhibition or not. Also, previously ETA has been shown to positively stimulate the autophagy [29]. In line with this observation, we performed a rescue experiment and allowed the wild‐type cells to grow in the presence or absence CAN 150 µm cosupplemented with or without 2.5 or 5 mm ETA for 12 and 18 h. The reason we chose to take these two time points (12 and 18 h) as opposed to previously used three (6, 12, 18 h) is because clear inhibition of autophagy was visible only after 12 and 18 h of treatment with higher doses. Our fluorescence microscopic analysis revealed that ETA supplementation induced autophagy by facilitating the delivery of GFP‐Atg8 fusion protein to the vacuoles marked by the GFP fluorescence signal inside the vacuoles as opposed to the GFP‐Atg8 cytosolic puncta seen in only CAN‐treated cells (Fig. 7A). We further validated our findings by performing GFP‐Atg8 processing assay by western blot analysis wherein we have shown that ETA supplementation resulted in the appearance of increased amount of free GFP, a direct readout of increased autophagic flux, as compared to only CAN‐treated cells (Fig. 7B,C). Next, we went on to check the levels of Atg8‐PE upon ETA supplementation [54]. Western blot analysis using anti‐Atg8 antibody revealed a significant increase in the level of Atg8‐PE as compared to Atg8 in ETA supplemented cells than only CAN‐treated ones (Fig. 7D,E). Together these findings suggest that exogenous supplementation with ETA remediated the CAN‐induced autophagy inhibition and hence cell viability.

### CAN likely perturbs autophagosome–vacuole fusion

**Fig. 8:**
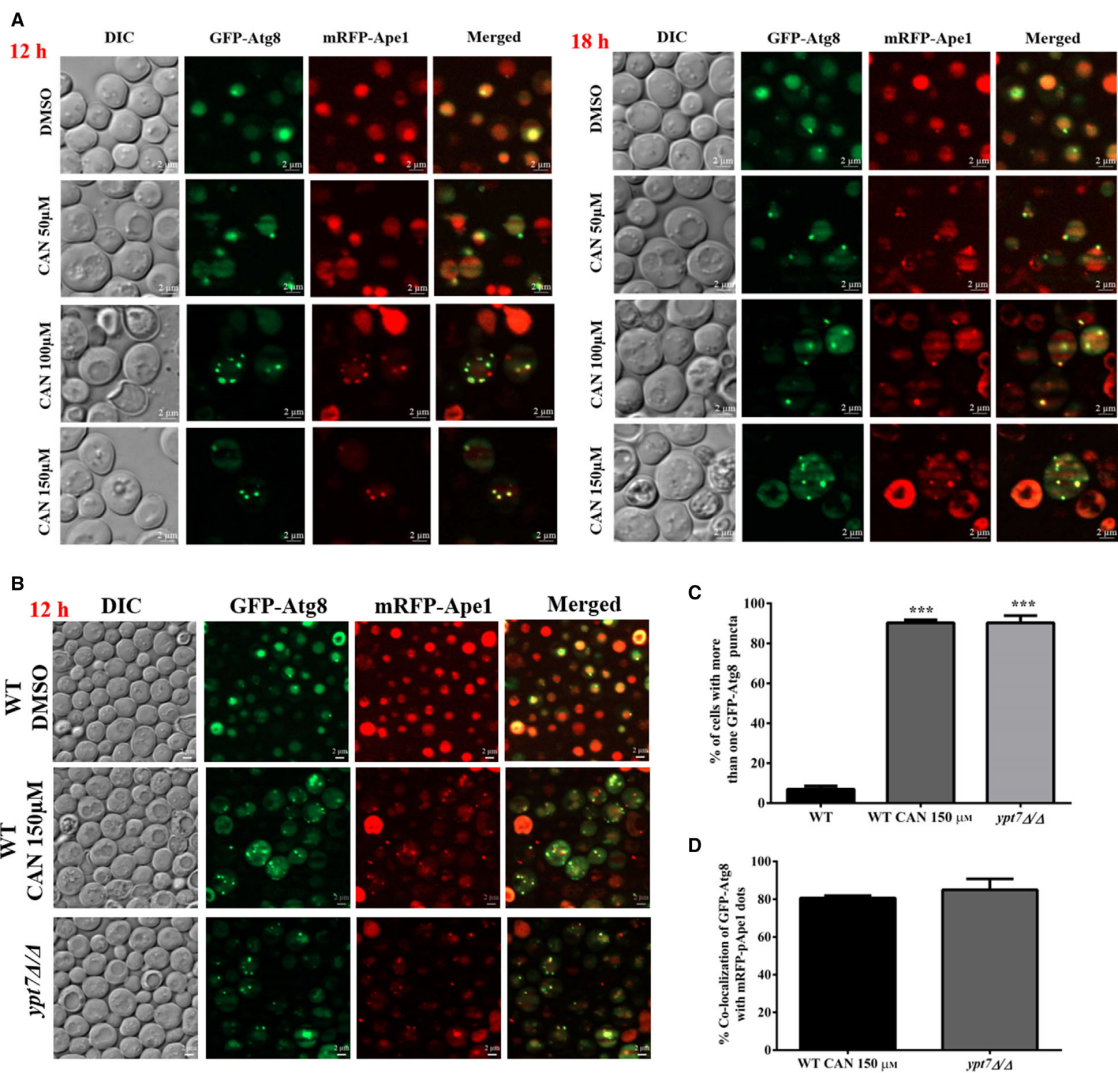
CAN likely perturbs the autophagosome–vacuole fusion. (A) Colocalization of GFP‐Atg8 and mRFP‐pApe1 dots. GYS638 cells harboring the GFP‐ATG8 plasmid were treated with indicated doses of CAN for 12 and 18 h. Post‐treatment cells were processed for fluorescence microscopic analysis using both red and green filters. Scale bar represents 2 μm. (B) Fluorescence microscopic analysis of GFP‐Atg8 and mRFP‐pApe1 in *ypt7Δ/Δ* untreated cells and in WT BY4741 cells treated with CAN 150 µm for 12 h. Scale bar represents 2 μm. (C) Quantification of percentage of cells containing more than one GFP‐Atg8 puncta. (D) Graph showing percentage of cells showing colocalization of GFP‐Atg8 with mRFP‐pApe1 dots. All data are representative of 3 biologically independent experiments and are presented as mean ± standard error mean (SEM). The significance of differences between sets of data was determined by Student’s *t*‐test; ns denotes *P* > 0.05, * denotes *P* ≤ 0.05, and ** denotes *P* ≤ 0.01 (as per the graph pad prism).

As CAN induced the formation of GFP‐Atg8 puncta, we wanted to ascertain its cytosolic localization. To check this, we determined the colocalization of GFP‐Atg8 with mRFP‐Ape1, a PAS, and autophagosome marker [55]. We observed that CAN treatment formed multiple GFP‐Atg8 dots and these dots very well colocalized with mRFP‐Ape1 dots (Fig. 8A). We next performed similar colocalization studies in both WT and autophagy mutant *ypt7Δ/Δ,* reported to be defective for autophagosome–vacuole fusion [55, 56]. We observed CAN‐induced accumulation of autophagosomes and this pattern is morphologically similar to that seen in *ypt7Δ/Δ* (Fig. 8B). ~ 90% of both CAN 150 µm treated and *ypt7Δ/Δ* cells contain more than one GFP‐Atg8 puncta and ~ 80% of these GFP‐Atg8 puncta colocalize with that of mRFP‐Ape1 dots (Fig. 8C,D). As Ypt7 is a Rab GTPase required for the fusion of autophagosomes to vacuole, CAN likely perturbs this crucial step required for completion of autophagy.

### Chloroquine sensitizes the autophagy inhibitory effects of CAN

**Fig. 9:**
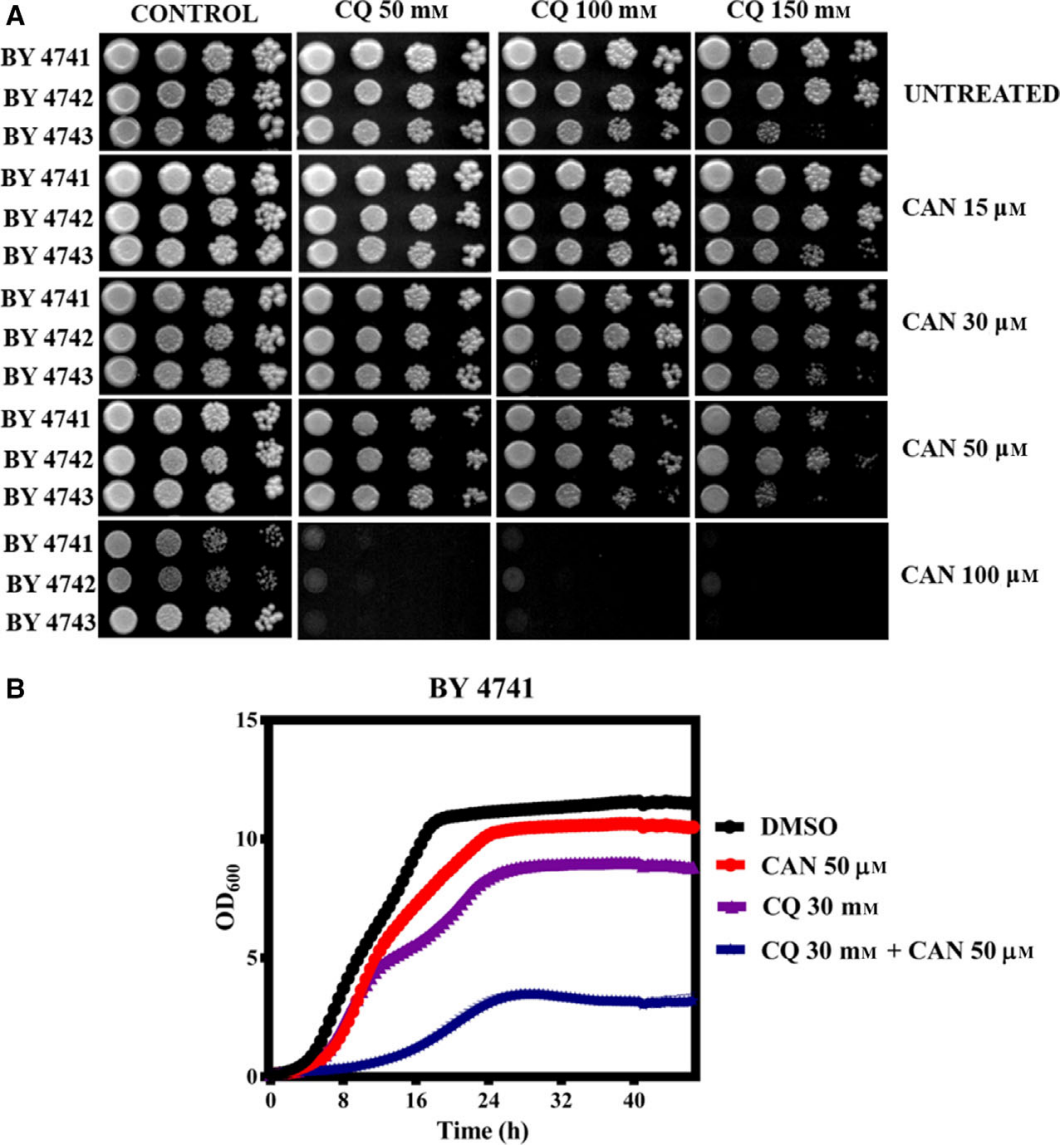
Chloroquine potentiates the autophagy inhibitory effects of CAN. (A) Yeast saturated cultures of wild‐type cells were 10‐fold serially diluted (10^−1^, 10^−2^, 10^−3^, 10^−4^) in 1.0 mL of sterile double‐distilled water and 3 µL of each dilution was spotted onto SCA plates containing DMSO (control) or sublethal doses of CAN (15, 30, 50, and 100 µm) or CQ (50, 100, and 150 µm) in alone or in different combinations as indicated. All plates were incubated at 30 °C for 72 h and scanned. (B) Growth curve assay: exponentially growing yeast cultures of the wild‐type strain BY4741 were treated with either DMSO (control) or indicated doses of CAN and CQ, and the growth was monitored by measuring OD_600_ at regular intervals of 30 min for ~ 44 h using a plate reader.

To further strengthen our findings that CAN inhibits autophagy, we performed a spot assay and liquid media growth curve analysis wherein we allowed the cells to grow in presence of sublethal dose of CAN and chloroquine (CQ). CQ mainly inhibits autophagy by impairing autophagosome fusion with lysosome [57, 58]. Several clinical trials have revealed that both chloroquine (CQ) and HCQ can sensitize the tumor cells and hence be used in combinatorial therapeutics [58]. Our hypothesis was if CAN is inhibiting autophagy and rendering the cells sensitive, then cotreatment with CQ should show hypersensitivity. Our data revealed that presence of CQ made the cells hypersensitive toward CAN (Fig. 9A,B). So CQ was able to sensitize the autophagy inhibitory effects of CAN.

## Discussion

CAN since the time of its discovery has been implicated as an anticancer molecule for its ability to inhibit PP2A, DNA repair proteins, cancer metastasis, etc. Though few genetic targets of this molecule are very well established, other potential genetic targets remain unexplored. Also, one study has reported CAN‐mediated autophagy inhibition [10]. However, which step of autophagy machinery is being targeted by CAN remains to be firmly demonstrated. Autophagy inhibition to treat cancer has been lately under extensive investigation as it enhances the efficacy of chemotherapeutics drug‐induced cell death [59]. Therefore, development of a new type of autophagy inhibitor for the treatment of cancer and understanding which stage of autophagy it blocks has important clinical significance [60, 61]. In the present study, in addition to identifying previously unexplored targets of CAN, we focussed on how CAN inhibits autophagy.

Initially, we performed spot assay analysis to determine the sublethal dose of CAN and found that CAN was able to inhibit the growth of wild‐type yeast cells in a dose‐dependent manner. We next investigated the molecular mechanism behind the CAN‐induced toxicity in yeast cells. As CAN is already reported to perturb the cellular lipid homeostasis, we first attempted to rescue the sensitive phenotype via supplementation of major phospholipid precursors like ETA, CHO, and INO. Only external supplementation of ETA, but not CHO or INO, drastically enhanced the yeast cell growth in presence of CAN. ETA when externally supplemented gets converted into PE inside the cells via the Kennedy pathway, and this PE is then added up to the total cellular PE pool which is then used by the cells for all downstream PE‐consuming pathways. We thus hypothesized that CAN might be targeting the cellular PE pool for exerting its toxicity as supplementation of ETA completely rescued this phenotype. One of the major contributors of cellular PE pool is Psd1, a mitochondrial residing enzyme catalyzing decarboxylation of PS to PE. So, gene expression analysis was done to study the levels of *PSD1* in presence and absence of CAN. As per our qRT‐PCR data, CAN decreased the *PSD1* expression in wild‐type yeast cells. This finding was further validated by performing growth phenotype assay in presence of both CAN and hydroxylamine hydrochloride (known to inhibit the decarboxylase activity of Psd1) which showed hypersensitive phenotype. Next, western blot experiment was done using anti‐Psd1 antibody where higher doses of CAN reduced Psd1 protein levels.

As CAN‐induced PSD1 downregulation, we next checked the effect of CAN on *psd1Δ* cells. We hypothesized that if CAN is downregulating *PSD1* expression, then deletion of PSD1 might render the cells hypersensitive. But to our surprise, the growth phenotype assays revealed that *psd1Δ* cells showed better tolerance toward CAN as compared to WT cells. We next went on to check the effect of CAN on cells overexpressing PSD1 and we observed that instead of showing rescue the cells were showing hypersensitive phenotype. From these findings, we concluded that downregulation of PSD1 expression might be a stress response of the yeast cells to tolerate CAN toxicity because of which *psd1Δ* cells tolerate CAN treatment better than the WT. Also, nonspecific inhibitors of PSD1 activity (NH_2_OH, DOX) may impair yeast survival by a PSD1‐independent mechanism.

To further strengthen our hypothesis, we examined the effect of CAN in downstream PE‐consuming pathways. Psd1 acts as a feeder enzyme for PE biosynthesis which then acts as a bottleneck for the three major downstream PE‐consuming pathways—membrane lipid integrity, PC synthesis, and autophagy. To check whether CAN perturbs membrane lipid integrity or not, we allowed the yeast cells to grow at 30 °C (permissive temperature for yeast cell growth) and 37 °C (nonpermissive temperature, induces heat stress) in presence and absence of CAN. Heat stress potentiated the toxic effects of CAN significantly, and this hypersensitivity could be rescued upon ETA supplementation.

Next, the effect of CAN in autophagy was examined by employing a well‐established autophagy marker GFP‐Atg8. Autophagy is an evolutionary conserved prosurvival strategy that the cell employs to mediate any external or internal stress by selective degradation of misfolded proteins or defective organelles so that their components can be recycled and reused. Atg8 is the major player in recruiting and directing the cargo for vacuolar delivery, degradation, and recycling. Atg8 is conjugated to PE, a lipidation process extremely important for autophagosome biogenesis and completion of autophagy. Our microscopic analysis using WT cells harboring the GFP‐ATG8 plasmid clearly showed the presence of GFP fluorescence in the form of cytosolic puncta in CAN‐treated cells, whereas the fluorescence shifted to vacuoles in untreated cells. This data thus revealed the failure of autophagy in CAN‐treated cells especially at higher doses. We further validated this finding by performing the western blot analysis using anti‐GFP antibody wherein we found the amount of free GFP band formed in case of CAN‐treated cells was significantly less as compared to untreated ones. Interestingly, increased Atg8 protein levels were also observed in CAN‐treated cells. One plausible explanation for this could be, as cells are exposed to CAN they are trying to endure this stress by upregulating Atg8 levels but they are failing to do so because of defect in vacuolar delivery of cargo. Our colocalization studies revealed CAN induced the accumulation of GFP‐Atg8 puncta which colocalizes with mRFP‐Ape1 dots. Also, the pattern of accumulation of autophagosomes is similar to *ypt7Δ/Δ* cells. Thus, we concluded that CAN is inhibiting autophagy flux probably by targeting the autophagosome–vacuole fusion leading to defects in vacuolar delivery of cargo. Furthermore, this CAN‐mediated autophagy inhibition could be completely rescued in presence of ETA as shown by the appearance of GFP fluorescence inside the vacuoles and increased amount of free GFP band. Supplementation of ETA has been reported to drive PE synthesis via the Kennedy pathway which in turn promotes fusion competence of the autophagosome and vacuole membrane.[62, 63]

Additionally, growing the cells in presence of sublethal doses of CAN and CQ showed hypersensitivity thus indicating the role of CQ in sensitizing the autophagy inhibitory effects of CAN. Autophagy inhibition has been widely accepted as a promising therapeutic strategy in cancer, while the lack of effective and specific autophagy inhibitors hinders its usage to treat tumors through its applications. At present, chloroquine (CQ) and its derivative hydroxychloroquine (HCQ) are only FDA‐approved drugs currently being used in patients as inhibitors of autophagy. Recent reports demonstrated the feasibility and beneficial effects of using CQ or HCQ, either alone or in combination in cancer therapy. Several studies indicate that autophagy acts as a positive regulator of tumor growth and survival in advanced cancers [59, 64, 65]. Tumors are exposed to extremely stressful conditions, including hypoxia and nutrient deprivation, and autophagy helps cells to overcome these stresses [66, 67]. In addition, autophagy fulfills the high metabolic and energetic demands of proliferating tumors by recycling intracellular components to supply metabolic substrates [66]. In animal studies, inhibition of autophagy or knockdown of autophagy genes results in tumor‐cell death [68]. In this line, further experiments need to be done in human cancer cell lines so as to ascertain the role of CAN as a potent autophagy inhibitor, one of the major survival strategies of cancer cells.

**Fig. 10:**
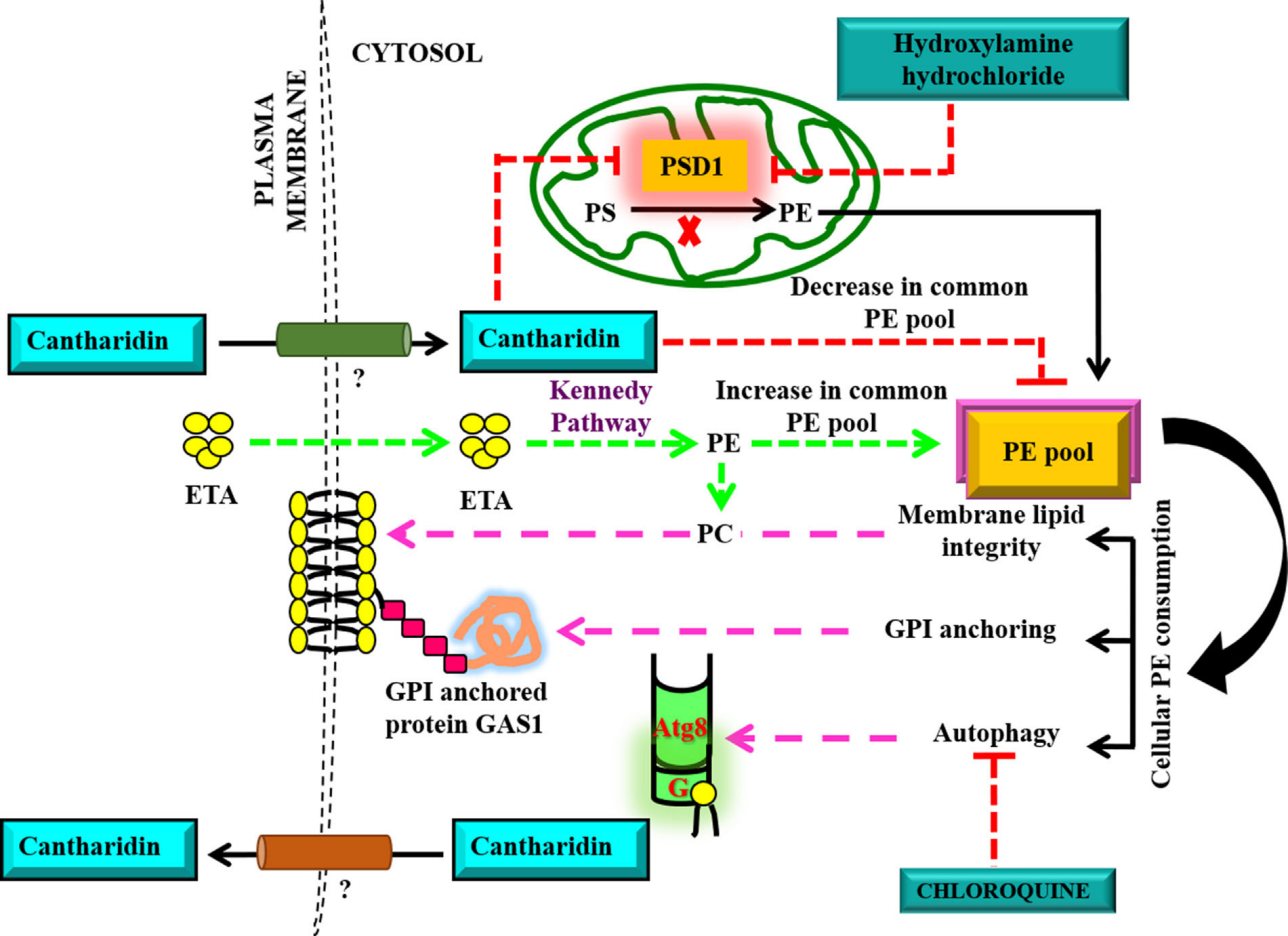
Proposed model for the mode of action of CAN. CAN upon its entry into the cell downregulates PSD1 expression thereby affecting downstream consuming pathways like membrane lipid integrity and autophagy. CAN also inhibits autophagy by targeting vacuolar delivery of cargos to vacuole. External supplementation of ETA bypasses the requirement of Psd1 enzyme and syntheses PE via Kennedy pathway thus restoring the downstream CAN‐affected pathways. In addition, hydroxylamine hydrochloride and chloroquine potentiated the cytotoxic effects of CAN.

Finally, we have proposed a model depicting the mode of action of CAN and possible pathways targeted by this anticancer molecule to exert its toxicity (Fig. 10). In summary, we have shown that CAN inhibits the expression of *PSD1* and probably affects the downstream PE‐consuming pathways. Although we have not measured PE directly, based on our current findings we believe that external supplementation of ETA bypasses the requirement of *PSD1* for de novo PE synthesis and uses the Kennedy pathway for probable restoration of PE pool back to normal levels and hence is able to rescue all observed CAN‐affected pathways. Notably, CAN inhibits autophagy. Also, as most of these pathways are highly conserved in higher eukaryotes, we believe that our findings in yeast could be translated to human cell lines as well.

## Conflict of interest

The authors declare no conflict of interest.

## Author contributions

SS and RST conceived and designed the project. SS performed all the experiments and analyzed the data. SS and RST interpreted the data and wrote the paper.

## Supporting information


**Fig. S1**. External administration of ETA rescues CAN‐mediated cytotoxicity.
**Fig. S2**. Cantharidin treatment upregulates *CRG1* expression.
**Fig. S3**. CAN inhibits PSD1 expression.
**Fig. S4**. *CRG1* or *PSD2* is not upregulated in psd1Δ cells.
**Fig. S5**. Autophagy in yeast.
**Fig. S6**. CAN inhibits autophagic flux.
**Table S1**. List of yeast strains used in the study.
**Table S2**. List of plasmids used in the study.
**Table S3**. List of primers used in the study.

## Data Availability

All supporting data are included in the manuscript as supplementary information.
