# Supplementary material for: Cantharidin downregulates PSD1 expression and inhibits autophagic flux in yeast cells
Source: FEBS Open Bio. 2022 Mar 29;12(5):1017–35. doi: 10.1002/2211-5463.13196 (PMC9063437; doi:10.1002/2211-5463.13196)
Supplement: Supplementary file 1 — Fig. S1. External administration of ETA rescues CAN‐mediated cytotoxicity. Fig. S2. Cantharidin treatment upregulates CRG1 expression. Fig. S3. CAN inhibits PSD1 expression. Fig. S4. CRG1 or PSD2 is not upregulated in psd1Δ cells. Fig. S5. Autophagy in yeast. Fig. S6. CAN inhibits autophagic flux. Table S1. List of yeast strains used in the study. Table S2. List of plasmids used in the study. Table S3. List of primers used in the study. [file FEB4-12-1017-s001.pdf]

Supplementary information

Cantharidin downregulates PSD1 expression and inhibits autophagic flux in yeast cells

Swati Swagatika[1] and Raghuvir Singh Tomar[1]*

[1]Laboratory of Chromatin Biology, Department of Biological Sciences, Indian Institute of Science Education and Research (IISER), Bhopal-462066, MP, India

Supplementary figures:

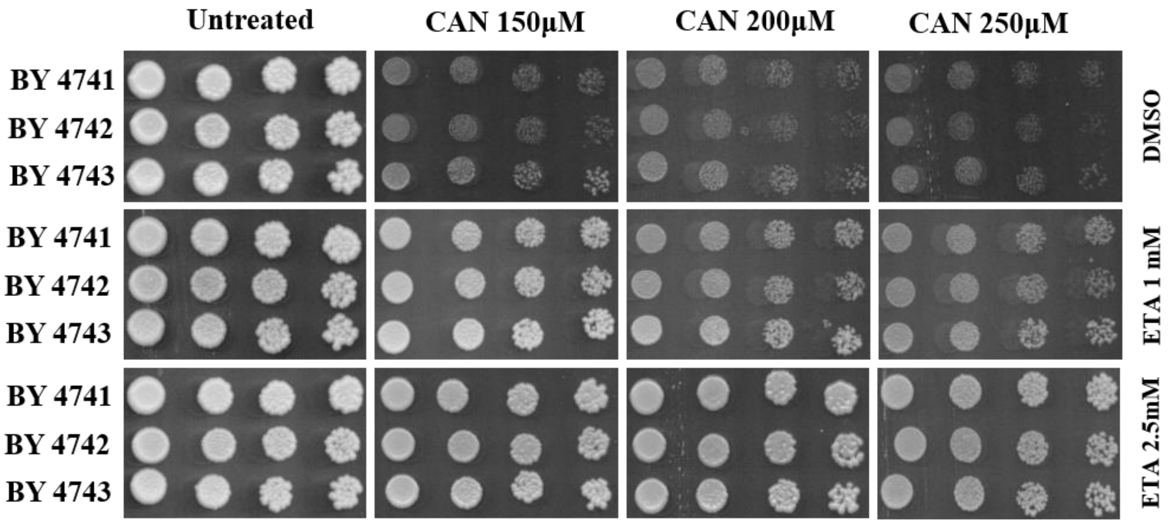

Figure S1. External administration of ETA rescues CAN mediated cytotoxicity. Serially diluted wild type yeast cells were spotted onto CAN containing plates supplemented with or without ETA 1 or 2 mM and images were scanned post 48h incubation at 30^0C.

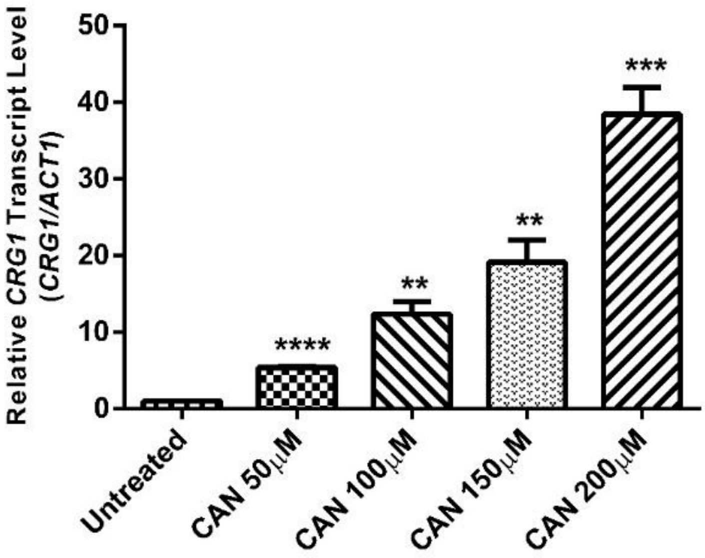

Figure S2. Cantharidin treatment upregulates *CRG1* expression. Real-time PCR (qPCR) quantification of CRG1 gene expression. Exponentially growing wild type yeast cells (BY4741) were treated with DMSO (control) or increasing doses of cantharidin (50, 100, 150 and 200 µM) and allowed to grow for 3h. After treatment, cells were harvested, RNA isolated and cDNA prepared. qPCR analysis of *CRG1* gene relative to the levels of *ACT1* mRNA was performed and values were plotted onto graph using graph pad prism software.

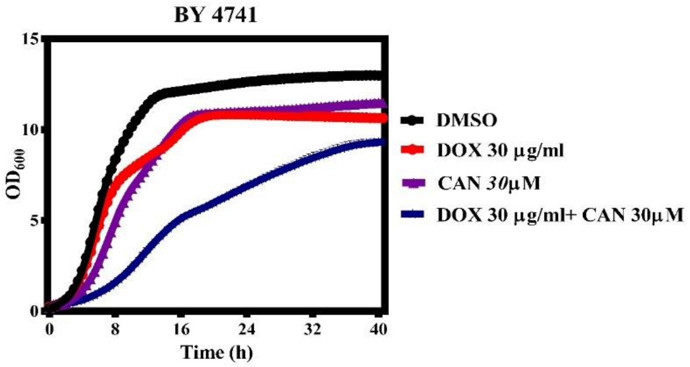

Figure S3. CAN inhibits PSD1 expression. Liquid media growth curve analysis was performed and exponentially growing WT cells were treated with indicated doses of either DOX or CAN or in combination and allowed to grow for 40h in an automated plate reader.

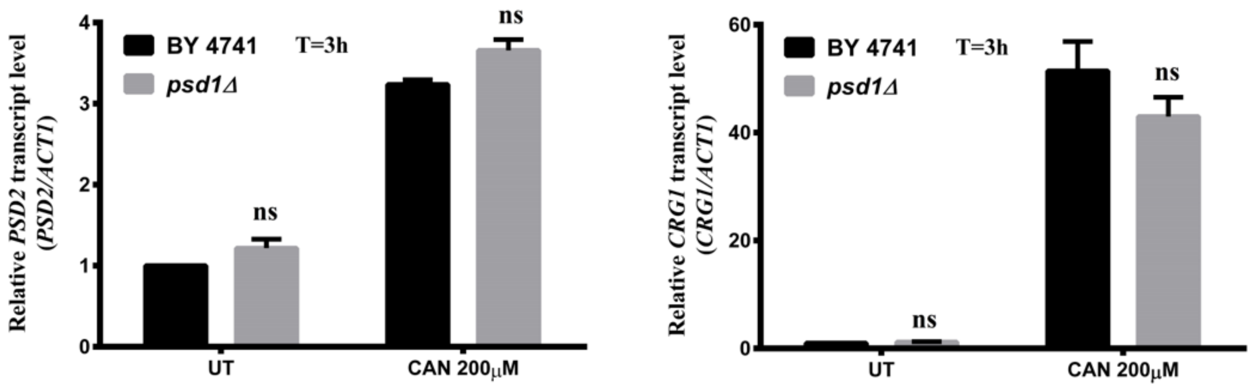

Figure S4. *CRG1* or *PSD2* is not upregulated in psd1Δ cells. qRT-PCR analysis was performed in order to compare the relative transcript levels of *CRG1* and *PSD2* in WT and *psd1Δ* cells before and after CAN treatment.

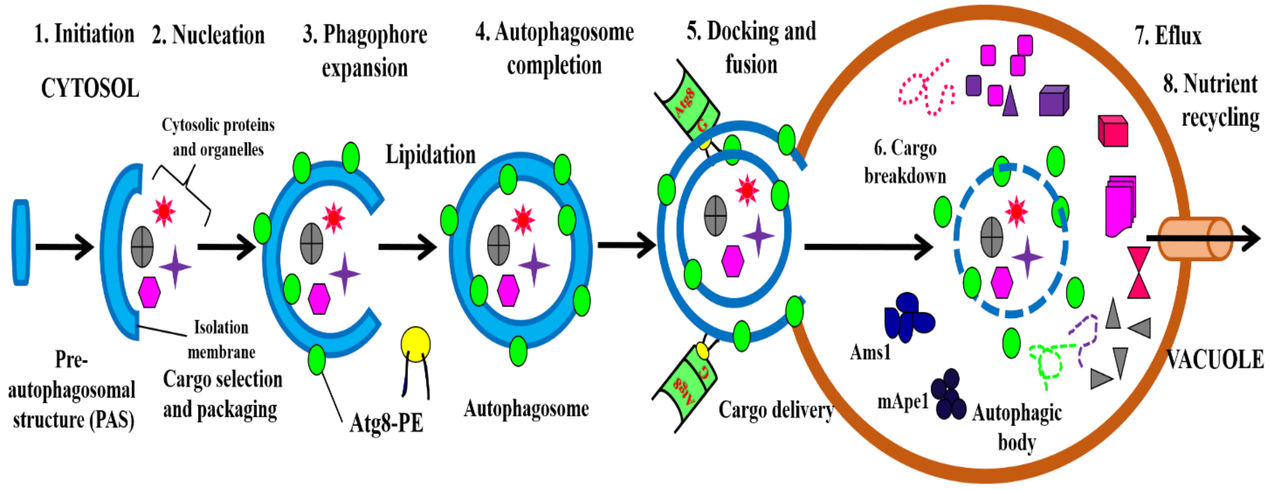

Figure S5. Autophagy in yeast. During autophagy cargo is sequestered into autophagosomes which upon vacuolar fusion releases autophagic body into its lumen. The cargo then undergoes vacuolar degradation by the action of vacuolar hydrolases in the lumen.

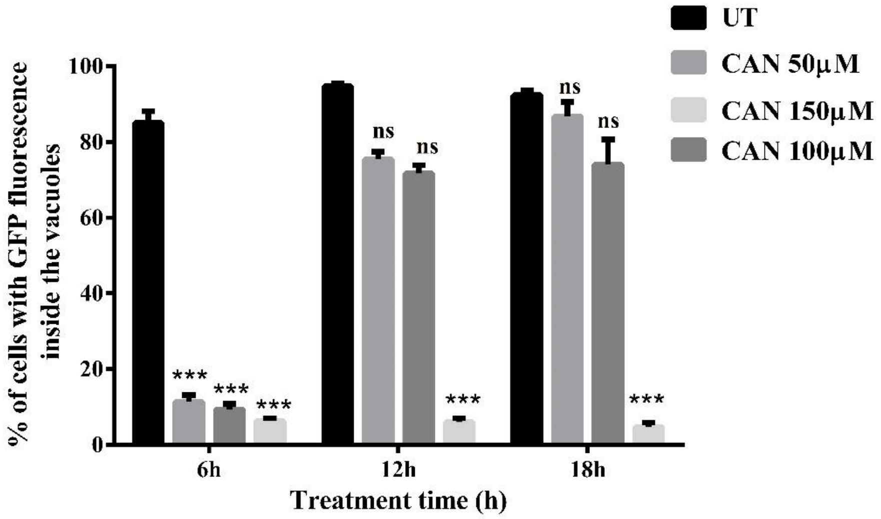

Figure S6. CAN inhibits autophagic flux. Quantification of percentage of cells with GFP fluorescence inside the vacuoles. 300 cells from each sample were counted.

Supplementary tables:

Table S1. List of yeast strains used in the study

Sl No.	Name of the yeast strain	Genotype	Source/ Reference
1	BY4741	MATa his3Δ1 leu2Δ0 met15Δ0 ura3Δ0	Yeast Knockout Collection-Open Biosystems (YKO-OB)
2	BY4742	MATα his3Δ1;leu2Δ0;lys2Δ0;ura3Δ0	YKO-OB
3	BY4743	MATa/α his3Δ1/his3Δ1 leu2Δ0/leu2Δ0 LYS2/lys2Δ0 met15Δ0/MET15 ura3Δ0/ura3Δ0	YKO-OB
4	*ypt7Δ/Δ*	BY4743, *ypt7Δ*::KanMX4	YKO-OB
5	*psd1 Δ*	BY4741, *psd1Δ*::HphMX4	[1]
6	*psd2Δ/Δ*	BY4743, *psd2Δ*::KanMX4	YKO-OB
7	Vph1-yEmCherry	Vph1- yEmCherry::HIS	[2]
8	GYS638	SEY6210; leu2::mRFP-APE1(LEU2)	[3]

Table S2. List of plasmids used in the study:

Sl.No.	Name of the plasmid used	Selectable marker	Source/Reference
1	CuGFP-ATG8(416)	Ura	Addgene

2	pRS425 Empty Vector	Leu	Dr. Yasushi Tamura’s lab [4]
3	pRS425 PSD1 2µ multicopy plasmid	Leu	Dr. Yasushi Tamura’s lab [4]

Table S3. List of primers used in the study

Sl No.	Name of the primer	Sequence
1	ACT1 FP	5’- TCGTCGGTAGACCAAGACAC-3’
2	ACT1 RP	5’- TTCTTCTGGGGCAACTCTCA- 3’
3	PSD1 FP	5’- CCGCTGAATGCGATGTCTCG- 3’
4	PSD1 RP	5’- GCTACTGGACGTGTGCCTG- 3’
7	CRG1 FP	5’- GGACTTTCCCGAAGCCTTGA- 3’
8	CRG1 RP	5’- GCATTTGGGTCCGAAGGACT- 3’
9	PSD2 FP	5’- TATACGACGCCCTACCAACCG- 3’
10	PSD2 RP	5’- GGCCGGTGTAGCTGAAGATG- 3’

Note: FP- Forward primer, RP- Reverse Primer

Supplementary references

1. Baker, C. D., Basu Ball, W., Pryce, E. N. & Gohil, V. M. (2016) Specific requirements of nonbilayer phospholipids in mitochondrial respiratory chain function and formation, *Molecular biology of the cell.* **27**, 2161-71.

2. Murley, A., Sarsam, R. D., Toulmay, A., Yamada, J., Prinz, W. A. & Nunnari, J. (2015) Ltc1 is an ER-localized sterol transporter and a component of ER-mitochondria and ER-vacuole contacts, *The Journal of cell biology.* **209**, 539-48.

3. Ngu, M., Hirata, E. & Suzuki, K. (2015) Visualization of Atg3 during autophagosome formation in Saccharomyces cerevisiae, *The Journal of biological chemistry.* **290**, 8146-53.

4. Tamura, Y., Onguka, O., Itoh, K., Endo, T., Iijima, M., Claypool, S. M. & Sesaki, H. (2012) Phosphatidylethanolamine biosynthesis in mitochondria: phosphatidylserine (PS) trafficking is independent of a PS decarboxylase and intermembrane space proteins UPS1P and UPS2P, *The Journal of biological chemistry.* **287**, 43961-71.
